# Supplementary material for: The interactome of intact mitochondria by cross-linking mass spectrometry provides evidence for coexisting respiratory supercomplexes
Source: Mol Cell Proteomics. 2017 Dec 8;17(2):216–32. doi: 10.1074/mcp.RA117.000470 (PMC5795388; doi:10.1074/mcp.RA117.000470)
Supplement: Supplemental Data [file supp_RA117.000470_133922_0_supp_23978_fzffwf.zip › spectra_annotation/mito_spectra_annotation/15-1-6-1-10-1.pdf]

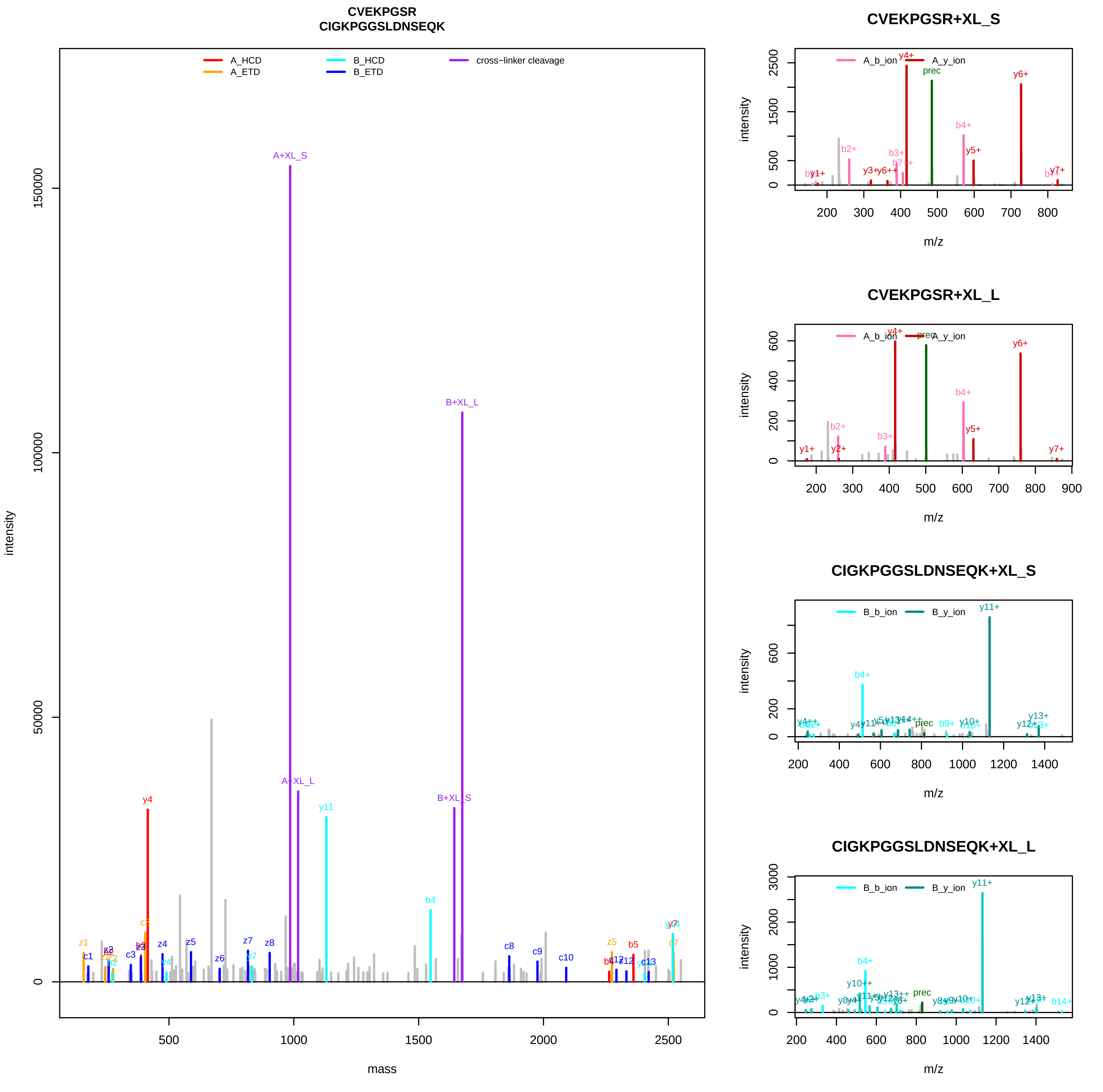

CVEKPGSR
CIGKPGGSLDNSEQK
A_HCD
A_ETD
B_HCD
B_ETD
cross−linker cleavage
A+XL_S
B+XL_L
A+XL_L
B+XL_S
y4
y11
b4
b5
y7
c7
z5
c3
c8
c9
c10
c13
z1
z4
z5
z6
z7
z8
c1
intensity
mass
0
50000
100000
150000
500
1000
1500
2000
2500
CVEKPGSR+XL_S
A_b_ion
A_y_ion
prec
y4+
y6+
b4+
y5+
b2+
b3+
intensity
m/z
CVEKPGSR+XL_L
A_b_ion
A_y_ion
y1+
y2+
y7+
intensity
m/z
CIGKPGGSLDNSEQK+XL_S
B_b_ion
B_y_ion
y11+
b4+
y13+
b9+
y10+
intensity
m/z
CIGKPGGSLDNSEQK+XL_L
B_b_ion
B_y_ion
y11+
b4+
y10++
b3+
b14+
intensity
m/z
